# Case Report: Death due to COVID-19 in Three Brothers

**DOI:** 10.4269/ajtmh.20-0240

**Published:** 2020-04-10

**Authors:** Sedigheh Yousefzadegan, Nima Rezaei

**Affiliations:** 1 Department of Pediatric Respiratory Medicine, Firouzabadi Hospital, School of Medicine, Iran University of Medical Sciences, Tehran, Iran;; 2 Research Center for Immunodeficiencies, Children’s Medical Center, Tehran University of Medical Sciences, Tehran, Iran;; 3 Department of Immunology, School of Medicine, Tehran University of Medical Sciences, Tehran, Iran;; 4 Network of Immunity in Infection, Malignancy and Autoimmunity (NIIMA), Universal Scientific Education and Research Network (USERN), Tehran, Iran

## Abstract

We report fatal cases of novel coronavirus disease (COVID-19) in three brothers in Iran. An increased susceptibility to specific pathogens has been reported for a number of genetic defects. Considering the fact that most of them who are affected by COVID-19 recover, deaths in three brothers who lived separately and had no known underlying disease suggest genetic predisposition to COVID-19 in some individuals.

Novel coronavirus disease (COVID-19), caused by infection with severe acute respiratory syndrome coronavirus-2 (SARS-CoV-2), was identified in late 2019 in Wuhan, China. It has rapidly spread around the world, and was named a pandemic by the WHO on March 11, 2020.^1,2^ In Iran, the first COVID-19 case was diagnosed on February 19, 2020; unfortunately, the Ministry of Health reports increasing numbers of cases and deaths from COVID-19.^3^

Interestingly, COVID-19 seems to be strangely and tragically selective. Only some infected people become sick. Although most critically ill COVID-19 patients are either elderly or have underlying medical problems such as cardiovascular disease, hypertension, diabetes mellitus, or cancer, some previously healthy and even relatively young individuals have died from COVID-19. Sequencing of patients’ genomes for DNA variations associated with severe illness may help to explain this mystery.^4^

Herein, we present the cases of three brothers, all of whom died from COVID-19 with a relatively similar pattern after less than 2 weeks of illness. The brothers, aged 54–66 years, lived in different locations in Tehran, Iran, and were previously healthy, without histories of underlying diseases, including hypertension, diabetes mellitus, cardiac or hepatic disease, or malignancy.

Patient 1, aged 60 years, worked in a travel agency. He referred to an outpatient clinic with fever, without any indication for admission. A dry cough started on the following day, followed by dyspnea 1 day later. He was then hospitalized with progression of dyspnea and suspicion for COVID-19. He was admitted to the intensive care unit (ICU) and died after 3 days with respiratory failure. A SARS-CoV-2 reverse transcriptase–polymerase chain reaction (PCR) test was positive; his spouse tested negative.

Patient 2, aged 54 years, worked in the same travel agency as his brother, case 1, with close contact with his brother beginning a week before the onset of illness. He started with fever 2 days after his brother. He was admitted promptly to another hospital. He developed cough and dyspnea 1 day later. The national treatment protocol for COVID-19 (hydroxychloroquine plus oseltamivir) was started. His condition improved by the fifth day of admission, and he was discharged from the hospital with O_2_ saturation of 87%. At home, despite good care, his condition deteriorated, and he died on the ninth day of illness. A SARS-CoV-2 reverse transcriptase–PCR test was negative, but the diagnosis of COVID-19 was highly suspected. SARS-CoV-2 tests for his spouse and their two daughters were negative.

Patient 3, aged 66 years, had only short contact for less than an hour with case 1 on his second day of illness. Patient 3 developed fever and cough 1 week later. He was admitted to a hospital with suspicion of COVID-19. He progressed to severe dyspnea 1 day later and, subsequently, was transferred to the ICU. He died on the next day with acute respiratory distress syndrome. A SARS-CoV-2 test was positive; tests were negative for his spouse and their two children.

The results of laboratory tests for the patients are presented in Table 1. Chest computed tomography scans are in Figure 1.

**Table 1:** Laboratory data of the patients

	Age (years)	C-reactive protein	White blood cell count (cells/µL)	Lymphocyte count (cells/µL; percentage of cells)	Novel coronavirus disease RT-PCR
Patient 1	60	248	9,100	910 (10)	+
Patient 2	54	102	6,000	420 (7.3)	−
Patient 3	66	113	4,100	615 (15)	+

**Figure 1.**
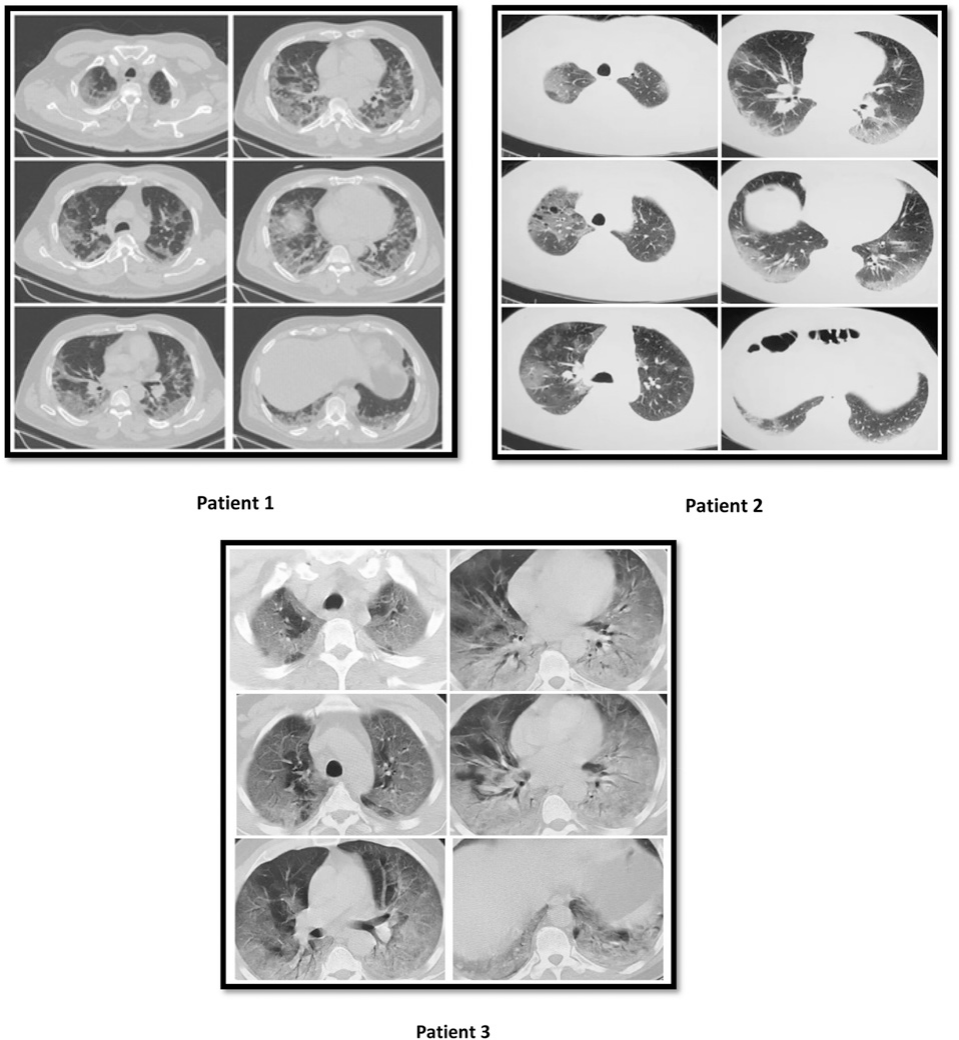
Chest computed tomography scans of patients 1, 2, and 3. All scans show diffuse ground glass opacities (GGOs), with peripheral distribution, consistent with novel coronavirus disease (COVID-19) lung involvement. Patients 1 and 2: crazy paving appearance, suggestive of late COVID-19. Patient 3: diffuse widespread GGOs and bilateral diffuse opacification due to acute respiratory distress syndrome, a severe complication of COVID-19.

There is some evidence for a role for the immune response and inflammation in the pathogenesis of COVID-19.^5^ An increased susceptibility to specific pathogens has been reported for a number of genetic defects.^6^ There have been reports of deaths from COVID-19 in multiple members of a family; for example, four family members died of COVID-19 in Wuhan, China, the epicenter of the outbreak, after they went into self-quarantine.^7^ Considering the fact that most of them who are affected by COVID-19 recover, death in three brothers who lived separately is of interest. Of note, the patients’ spouses and children were not shown to be affected by COVID-19, with negative tests for SARS-CoV-2. Therefore, the three brothers who experienced fatal COVID-19 may have had a particular predisposition to infection or severe illness. It can be hypothesized that there might be a genetic predisposition to COVID-19 and/or to severe illness from COVID-19 that renders some individuals without any underlying disease at particular risk of severe or fatal illness.
